# Acoustic emission characteristic of sandstone and sandstone like material under multi-path loading

**DOI:** 10.1371/journal.pone.0297087

**Published:** 2024-01-25

**Authors:** Tong Zhuang, Rui Peng, Qifeng Zhao, Shiyuan Jiang, Xuehong Yang, Chunyang Shen, Jiang Liu

**Affiliations:** 1 School of Mine Safety, North China Institute of Science and Technology, Beijing, China; 2 School of Safety Engineering, North China Institute of Science and Technology, Beijing, China; Sapienza University of Rome: Universita degli Studi di Roma La Sapienza, ITALY

## Abstract

Using spline interpolation to select proportions of similar materials, a comparative analysis of the fracturing behavior of sandstone specimens and similar material specimens was conducted through Brazilian splitting tests under multi-path loading. The study revealed that during stepwise loading, both sandstone and similar materials exhibited memory effects and plastic deformation. However, under constant velocity loading, the relationship between force and displacement in sandstone showed linearity after compaction. Employing MATLAB optimization algorithms for the inversion of acoustic emission event information, the distribution of fracture points, and the evolution of cracks were analyzed. The findings indicated that under stepwise loading, both sandstone and similar materials exhibited banded distribution of peak frequencies, with sandstone concentrated in the mid-low-frequency range and similar materials leaning towards the low-frequency range. The amplitude-frequency characteristics of acoustic emission signals suggested that initially, sandstone produced low-frequency, low-amplitude signals. As cracks developed, these signals gradually transformed into high-frequency, high-amplitude signals, ultimately leading to macroscopic failure. The ringing counts and b-values of sandstone displayed an approximate "W" shape distribution, with a subsequent decrease in b-values during final failure. In contrast, the acoustic emission counts were inversely related to b-values. Similar materials exhibited slightly more acoustic emission counts than sandstone, with relatively lower b-values. The crack development process of both sandstone and similar materials was confirmed through these observations. From the perspective of section initiation and local damage, sandstone and similar materials exhibited similar failure characteristics. The proportions of quartz sand: cement: water = 9:1:0.9 in similar materials demonstrated the most similar characteristics to sandstone in terms of mechanical loading, acoustic emission features, and failure morphology. This suggests that these similar materials can be used as substitutes for sandstone in analogous simulation experiments. The study provides theoretical support for understanding rock fracture mechanisms, offers guidance for the selection and proportioning of similar materials, and holds significance for predicting and controlling rock fracture behavior in engineering applications.

## 1 Introduction

The advancement of detection technology has increasingly attracted scholars to apply it in rock mechanics engineering research. Techniques such as acoustic emission, infrared remote sensing, digital speckle, CT scanning, electron microscopy scanning, and others have all seen successful applications [1–8]. Acoustic emission involves using specialized equipment to monitor and record the sound waves produced by microcracks or displacement released internally in rocks due to stress concentration during loading. By monitoring acoustic emission information, it is possible to understand changes in rocks during the loading process, such as the formation, propagation, and failure of cracks. Acoustic emission is characterized by its real-time nature, rich data information, and high efficiency. Existing research indicates a close correlation between the acoustic emission characteristics of rocks and their stress states. Therefore, acoustic emission detection technology can greatly help reveal the mechanisms and mechanisms of rock deformation and failure.

Many scholars have observed a close connection between the characteristic parameters of acoustic emission (AE) signals and the deformation and failure characteristics of rocks. Zhao et al. [9] through assessing the AE characteristics of sandstone under triaxial compression, revealed the relationship between the damage process and the dynamic phenomena in rocks (rapid changes in stress during the loading process). Zhang et al. [10] conducted direct tensile tests, studying the spatial and temporal distribution of AE signals throughout the loading process. They analyzed the distribution and evolution patterns of the normalized applied stress in AE waveform main frequencies. Zhao et al. [11] discussed the relationship between the loading stress corresponding to the minimum average frequency centroid of AE signals and the peak stress during the deformation and failure process of red sandstone specimens. Zhang et al. [12] concluded through their study that the variation pattern of AE energy is consistent with the rock’s damage process in sandstone. In order to uncover the spatiotemporal correspondence between acoustic emission event signals and the generation and development of rock fractures, scholars have conducted in-depth research on the signals of acoustic emission events and the characteristics of rock damage. Feng et al. [13] utilized AE monitoring to unveil the spatiotemporal evolution patterns of rock damage. Li et al. [14] used AE monitoring to study AE event activity during the damage stage, further analyzing the spatiotemporal evolution patterns of materials. Wu et al. [15] conducted research based on Brazilian splitting tests to investigate the correspondence between granite AE event location and damage. Zha et al. [16] analyzed the spatiotemporal evolution of AE parameters and rock damage development under different loading modes. Li et al. [17] established, through experiments, the relationship between the frequency of rock fracture AE signals and crack scale. Zhao and Liu [18] discovered that the cumulative AE energy suddenly increases before reaching peak strength, correlating positively with the development of penetrating cracks. Zhao et al. [19] obtained AE parameters and typical modes for different fracture types, discussing the energy release characteristics of each fracture mechanism. Lei et al. [20] through a series of loading and unloading experiments on surrounding rocks, found that the spatial distribution of AE events is related to the stress state and structure of the specimens.

Scholars have extensively researched the acoustic emission characteristics of loaded rocks, yielding numerous valuable findings, especially concerning the correspondence between acoustic emission signals and rock deformation and failure. However, much of the existing research is specific to particular engineering projects, involving specimens with regional characteristics. Consequently, the conclusions drawn might not be universally applicable to other areas. Hence, scholars increasingly prefer selecting universally applicable rock-like materials for corresponding studies in rock mechanics. Utilizing spline interpolation, a reasonable mix of rock-like materials akin to sandstone was chosen, subjecting both sandstone and these rock-like materials to Brazilian splitting experiments under different loading conditions. Analysis of the mechanical failure characteristics of specimens and comparison of the similarities in failure between sandstone and rock-like materials were conducted. Acoustic emission signals were collected for feature analysis, and MATLAB optimization algorithms were employed to explore the spatiotemporal evolution patterns. The research findings offer theoretical support for revealing rock fracture mechanisms and selecting similar material compositions.

## 2 Engineering background and test process

### 2.1 Engineering background

Damage caused by tensile stress is one of the common problems in underground space engineering, often encountered in projects such as tunnels, underground chambers, and coal mining. This is due to the fact that rocks are resistant to compression but not to tension. Due to the complex geological conditions and working environments in underground engineering, conducting in-situ tests to observe and measure the damage caused by tensile stress is often extremely challenging. Therefore, similar simulation experiments have become a primary means to explain and study the mechanism of damage caused by tensile stress.

Similar simulation experiments involve constructing scaled-down models, using similar materials and loads to simulate the process of tensile stress-induced damage in real engineering projects. This testing method can provide information about the form, process, and mechanism of damage. However, similar simulation experiments currently face several major challenges, one of which is the selection and corresponding ratio of similar materials. The similar materials used in these experiments should possess physical and mechanical properties similar to those of actual engineering materials to ensure the reliability and repeatability of the test results. However, different scholars have differences in the selection and ratio of similar materials, and a unified scientific understanding has not yet been established.

**Fig 1:**
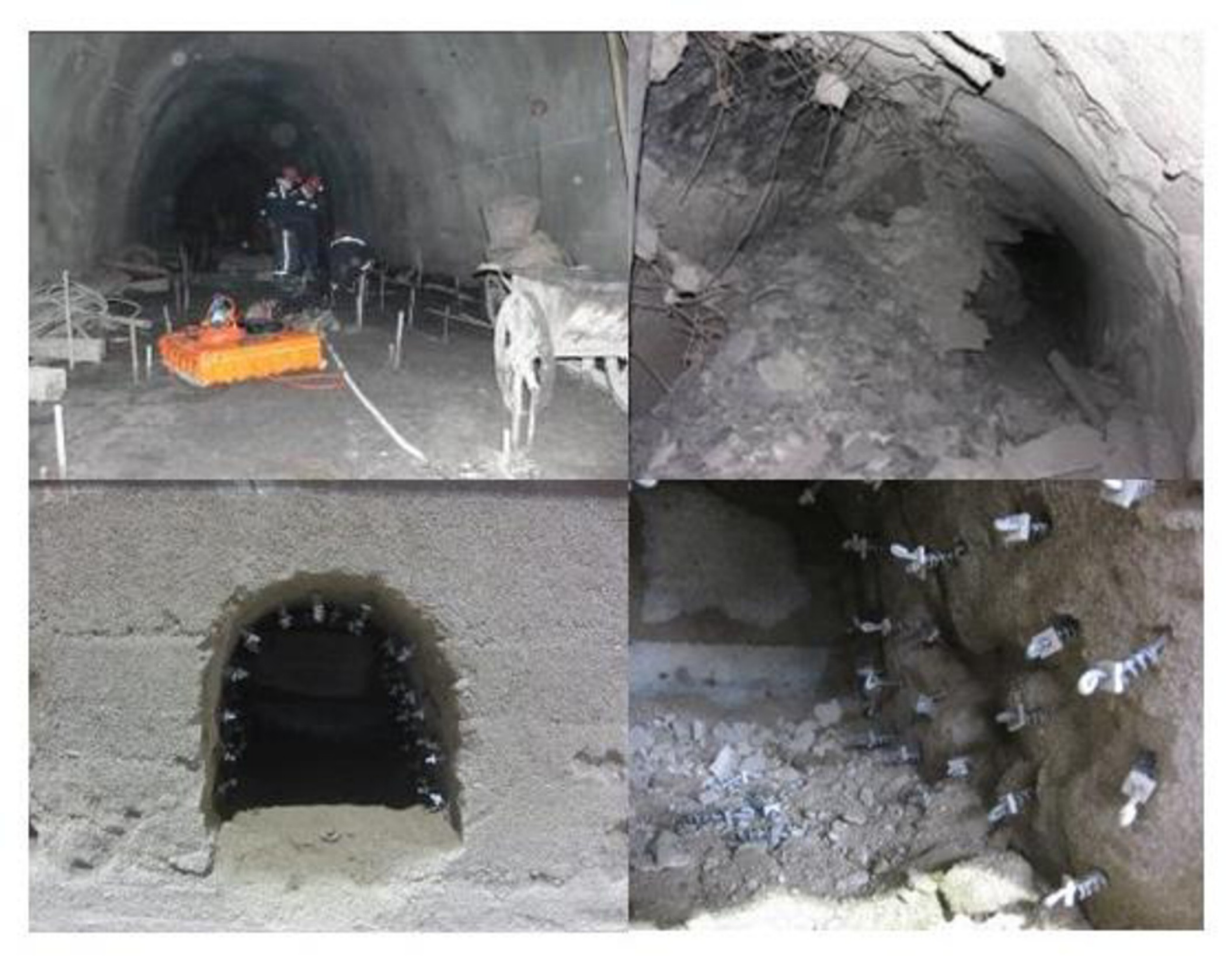
Comparison of roadway and similar model before and after tensile failure.

It has been thoroughly proven through indoor research that rocks are resistant to compression but not to tension. Meanwhile, scholars have proposed that the phenomenon of tensile damage has become a major cause of rock tunnel damage. As shown in Fig 1, a typical damage phenomenon occurred in a mining excavation tunnel under tensile stress, and similar simulation experiments were conducted to obtain similar damage structures. This study plans to conduct in-depth research on the causes of damage caused by tensile stress through the results of similar materials under tensile stress. It aims to summarize the experience of selecting similar materials, providing a scientific basis for similar simulation experiments on tensile stress damage in underground space engineering, and further advancing the understanding and predictive capabilities of damage mechanisms.

**Fig 2:**
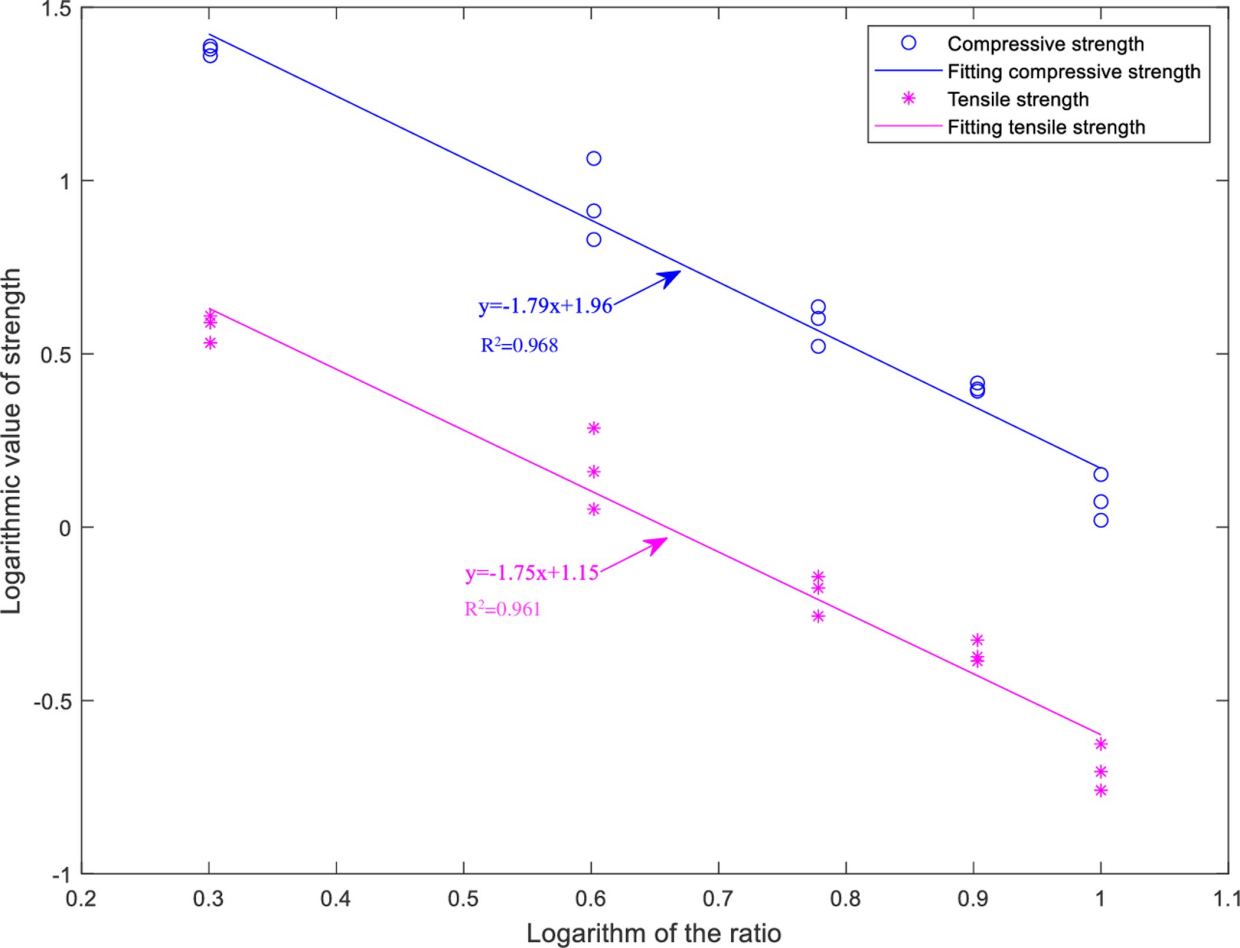
Double logarithmic fitting diagram of ratio and strength.

In the previous research [21], physical and mechanical tests were conducted on similar materials composed of sandstone specimens and a mixture of quartz sand (aggregate), cement (binder), and water with different ratios. The results are shown in Tables 1 and 2. Analysis of the relationship between sand-to-cement ratio and compressive strength, as well as tensile strength, revealed a linear relationship in the logarithmic scale, as depicted in Fig 2. It can be observed that as the sand-to-cement ratio increases, both compressive strength and tensile strength exhibit a decreasing trend.

**Table 1:** Physical performance parameters of rock samples.

lithologic characters	elastic modulus (GPa)	deformation modulus (GPa)	compressive strength (MPa)	tensile strength (MPa)	poisson ratio
coarse sandstone	5.56	6.42	35.02	5.64	0.21

**Table 2:** Mechanical performance parameters of similar materials with different proportions.

Group name	mechanical parameter	Similar material ratio, aggregate: cement: water				
		2:1:0.2	4:1:0.4	6:1:0.6	8:1:0.8	10:1:1
Group 1	compressive strength (MPa)	24.426	6.757	3.325	2.468	1.047
	elastic modulus(GPa)	0.247	0.815	0.342	0.746	0.577
	deformation modulus (GPa)	0.321	1.401	0.741	2.431	0.353
	tensile strength(MPa)	4.069	1.127	0.554	0.411	0.174
Group 2	compressive strength (MPa)	22.904	8.171	4.001	2.504	1.185
	elastic modulus(GPa)	0.227	0.558	0.530	0.215	0.415
	deformation modulus (GPa)	0.300	0.563	0.591	0.484	0.565
	tensile strength(MPa)	3.891	1.445	0.667	0.422	0.197
Group 3	compressive strength (MPa)	23.925	11.579	4.324	2.606	1.419
	elastic modulus(GPa)	0.210	0.446	0.501	0.249	0.415
	deformation modulus (GPa)	0.260	0.512	0.583	0.554	0.465
	tensile strength(MPa)	3.403	1.930	0.720	0.472	0.237

In this study, it is planned to create similar simulation specimens with a stress similarity ratio of 20 to simulate the tunnel prototype. The surrounding rock of the tunnel is predominantly coarse sandstone, and the tensile strength is 5.64 Mpa, so the corresponding tensile strength of the similar material is 0.28 MPa. Considering the logarithmic linear relationship between sand-to-cement ratio and strength, the corresponding sand-to-cement ratio is approximately 9:1. To facilitate the experimental research on proportioning, three ratios of 10:1, 9:1, and 8:1 were selected according to the standards for specimen preparation. These choices will help determine the optimal proportioning for constructing the similar model.

### 2.2 Sample preparation

The test specimens used in the experiment were extracted from both sidewalls of a mine tunnel. To ensure consistent mechanical performance of the specimens, all specimens were obtained from the same rock block without prominent jointing. Following the recommendations of the International Society for Rock Mechanics, standard specimens with a height of 25mm and a diameter of 50mm were prepared.

For the similar material, a mixture of washed quartz sand, Portland cement, and pure water was poured in different ratios: 10:1:1 (ratio 1), 9:1:0.9 (ratio 2), and 8:1:0.8 (ratio 3). These mixtures were used to prepare standard specimens with a height of 25mm and a diameter of 50mm.

**Fig 3:**
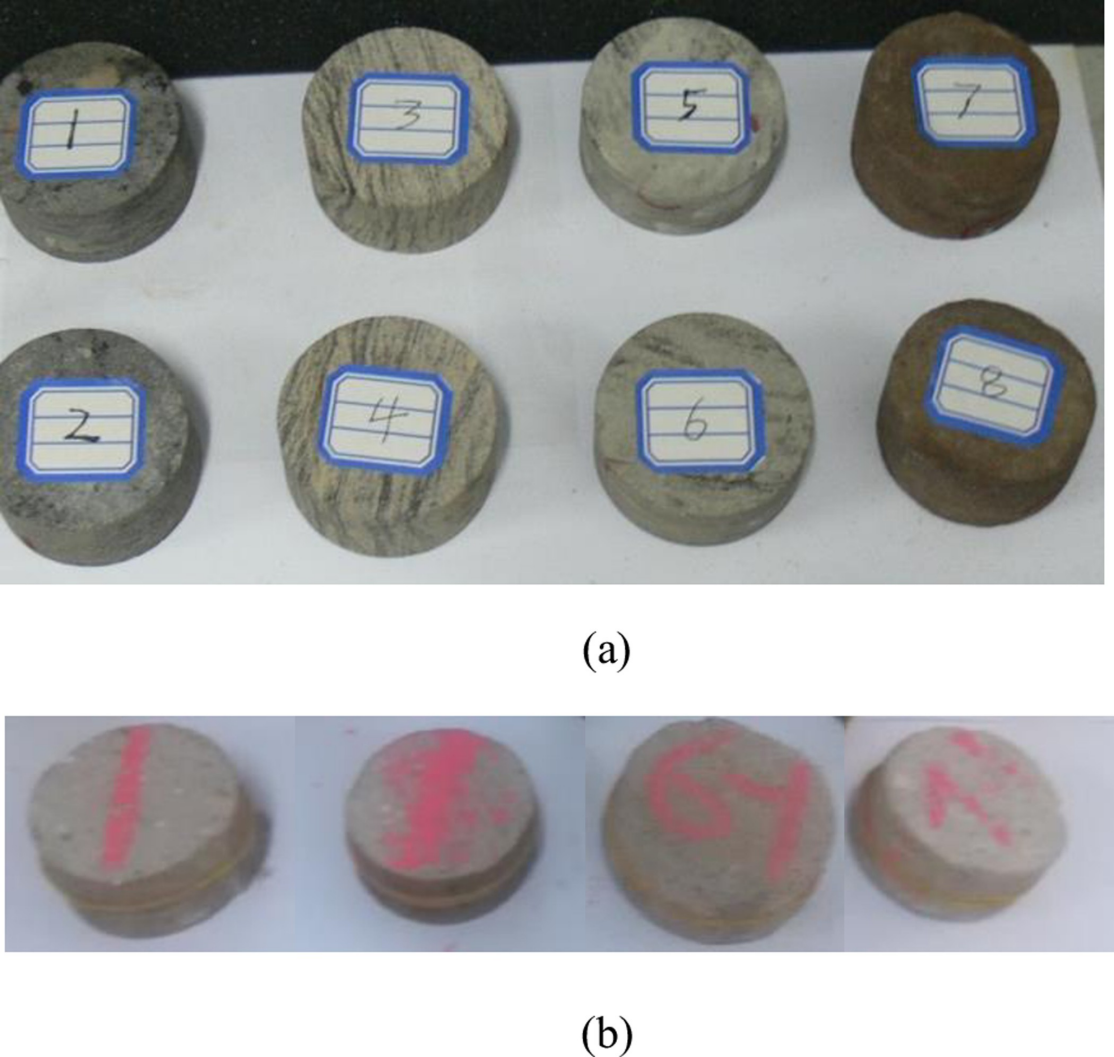
Part of the test block picture: (a) sandstone (b) similar material.

Some test blocks are shown in Fig 3.

### 2.3 Test loading equipment

The experiments were conducted using the MTS-816 rock testing system, which consists of components such as the MTS system, load frame, motor, pressure gauge, displacement transducer, and strain gauges. This system can simulate various load-deformation conditions, with a maximum load capacity of ±125kN, load resolution of 1N, maximum displacement of ±25mm, and deformation resolution of 0.0001mm. It also records data during testing to evaluate the mechanical properties of the sample, such as strength, modulus, creep behavior, etc. The MTS-816 system offers several functionalities and advantages, including high precision and repeatability, wide applicability, ease of use, and adjustable factors for samples. It can perform various tests such as compression, tension, dynamic loading, consolidation, and stress path tests. As a result, the MTS-816 rock testing system is widely applied in fields such as geotechnical engineering and mining engineering to assess the properties and behavior of rocks. In addition, by connecting to a computer, real-time monitoring and updating of experimental status data are possible, further expanding the application range of this testing system.

In this study, the Brazilian tensile test on sandstone was conducted using two loading methods. One method involved a force-controlled mode with a constant loading rate of 0.02kN/s until the specimen fractured. Another kind of loading adopts the force control mode to carry out the variable lower limit step loading. The loading and unloading are carried out at a constant speed of 0.02 kN / s, and the loading is carried out step by step until the test block is destroyed.

### 2.4 Acoustic emission monitoring

**Fig 4:**
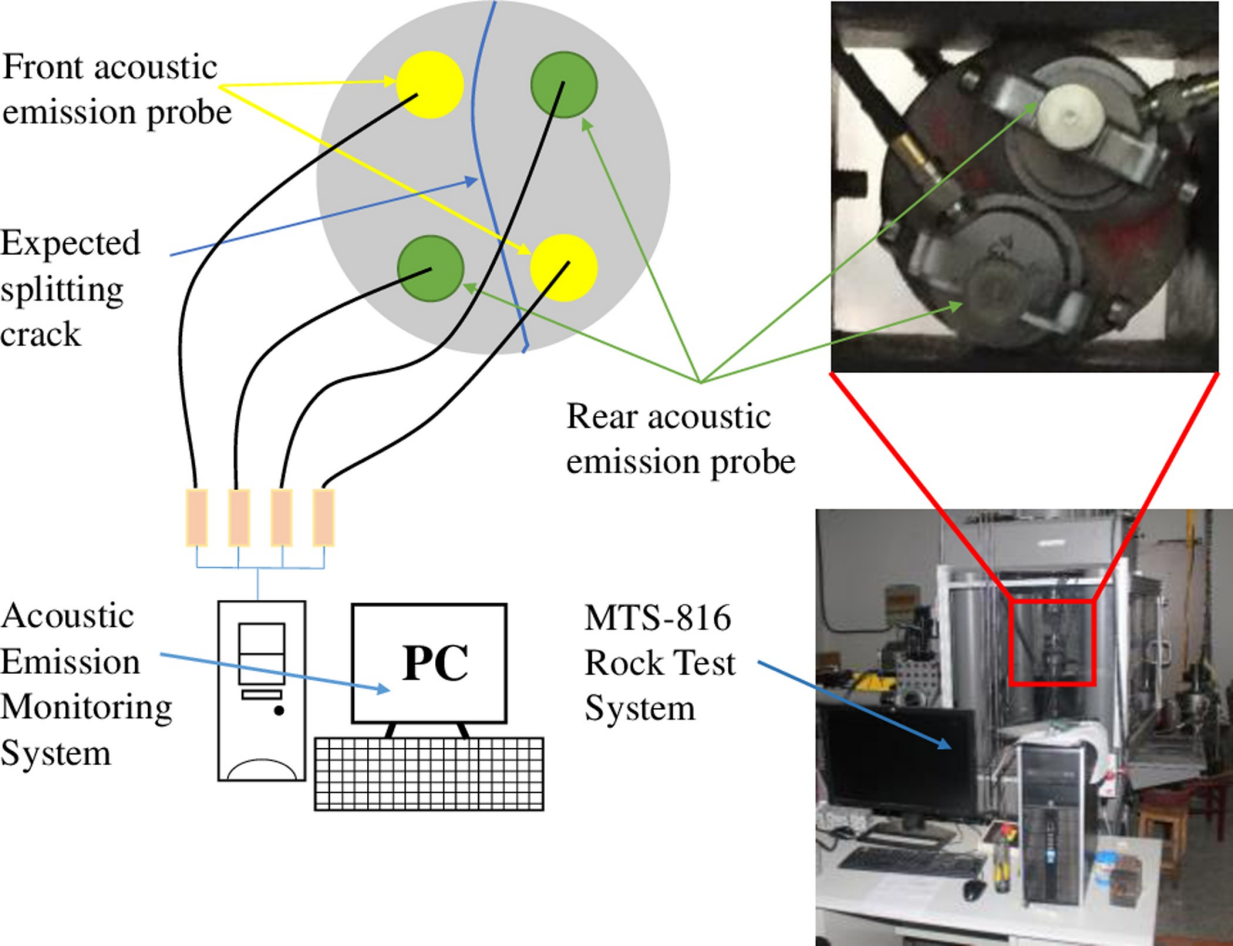
MTS-816 and acoustic emission monitoring system.

During the experiment, two acoustic emission sensors were used on each circular surface for monitoring acoustic emission events, as shown in Fig 4. The sensors were arranged radially in a direction at a 45° angle to the horizontal, ensuring that two sensors remained in monitoring position on each fractured segment of the specimen. This provided more accurate data support for the inversion of the event points after fracturing. To ensure a tight fit between the sensors and the specimen, high-strength epoxy resin adhesive was used to secure the sensor brackets to the specimen, and petroleum jelly was applied to the acoustic emission sensors to ensure sufficient coupling.

## 3 Results and analysis

### 3.1 Force and displacement

**Fig 5:**
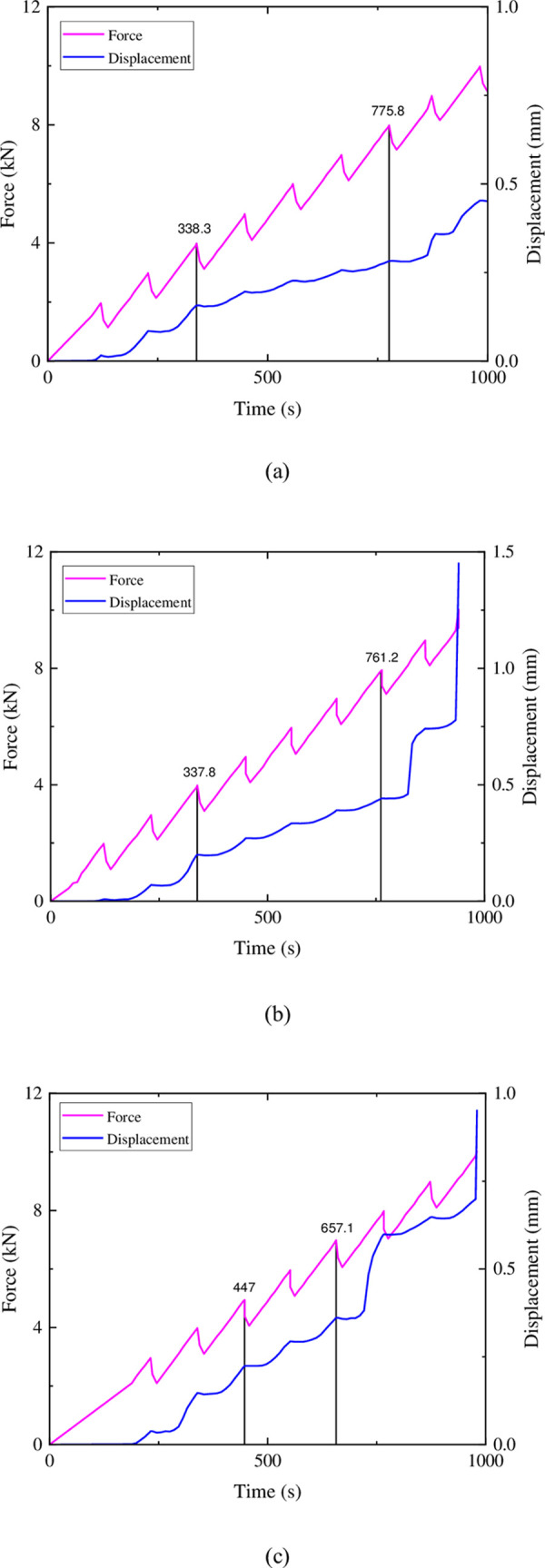
Force and displacement in sandstone variable lower limit step loading.

In the Brazilian tensile test with incremental loading and unloading on sandstone, the first loading increment reached 2kN and then was unloaded to 1kN. The second loading increment reached 3kN and then was unloaded to 2kN. Each subsequent loading increment and unloading limit increased by 1kN compared to the previous increment, until the completion of the test. The relationship between force and displacement during the incremental loading is shown in Fig 5.

It can be observed that during the initial stage of loading (0–338.3s in Fig 5(A), 0–337.8s in Fig 5(B), 0-447s in Fig 5(C)), the displacement is essentially zero in the first loading increment. However, it rapidly increases from zero during the second and third loading increments. In the subsequent loading increments (338.3–775.8s in Fig 5(A), 337.8–761.2s in Fig 5(B), 447–657.1s in Fig 5(C)), the rate of displacement increase slows down. In the 2–4 loading increments leading up to the peak force, the displacement rate increases again, surpassing the initial stage. Additionally, except for the first loading increment, during each loading process, the displacement remains relatively constant from the unloading of the previous increment to the peak force of the current increment. When the force of the current increment surpasses the peak force of the previous increment, the displacement continues to increase with the increasing force. This phenomenon is similar to the memory effect observed in rock specimens during incremental uniaxial compression tests, indicating the presence of memory in rocks. However, unlike in compression tests, the deformation does not recover with the unloading of the force. In the Brazilian tensile test, the deformation observed in the rocks is predominantly plastic deformation.

**Fig 6:**
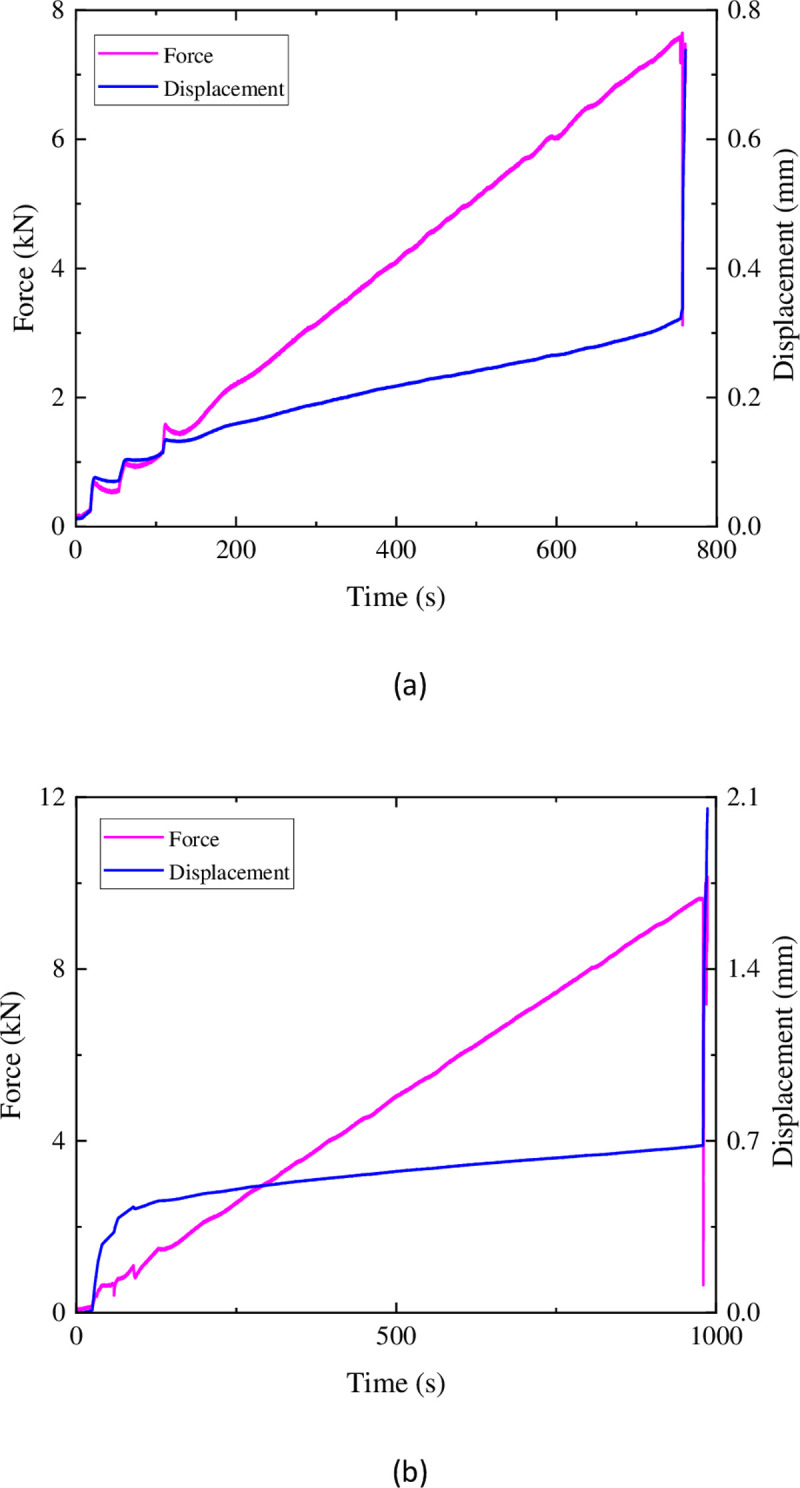
Force and displacement in sandstone uniform loading.

In the constant-rate loading Brazilian tensile test on sandstone, as shown in Fig 6(A), during the initial stage (0s-120s), both displacement and force exhibit fluctuations. It is inferred that during this stage, with the loading of the force, localized stress concentration occurs at the steel wire, leading to a cycle of localized failure, equilibrium, and subsequent failure of the specimen, ultimately establishing a new equilibrium. As localized failure of the specimen occurs, there is a sudden change in displacement, followed by a gradual increase as the new equilibrium is established. After 2–3 cycles, the localization becomes compacted, and the force and displacement exhibit an approximate linear relationship. A linear regression analysis indicates a slope of 30.85, intercept of -2.72, and an R^2^ value of 0.988. As loading continues until reaching the tensile strength of the specimen, the specimen experiences complete failure, and the displacement exhibits a linear increase at the moment of failure.

In Fig 6(B), during loading up to 25s, the displacement rapidly increases from 0mm to 0.4mm. Subsequently, similar to Fig 6(A), the displacement shows an approximate linear relationship with the force. Linear regression analysis yields a slope of 29.86, intercept of -12.01, and an R^2^ value of 0.988. This linear relationship persists until the failure of the specimen.

**Fig 7:**
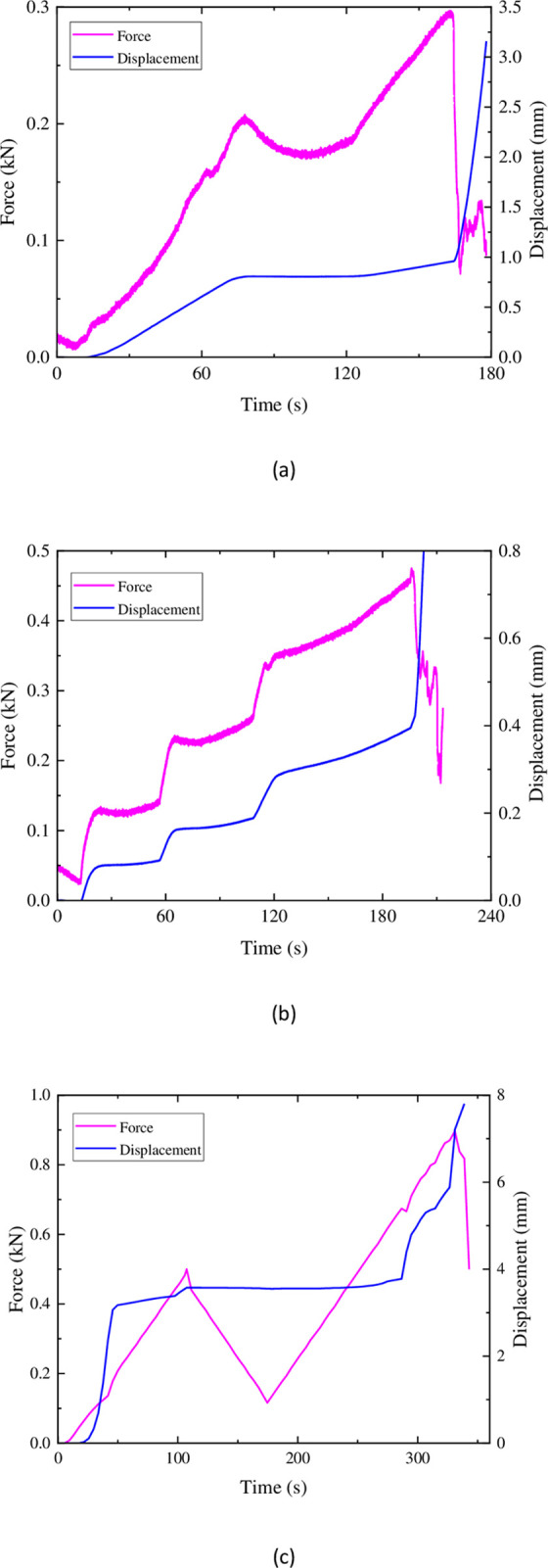
Force and displacement in variable lower limit step loading of similar materials: (a) ratio 1 (b) ratio 2 (c) ratio 3.

Cascade loading of similar materials is shown in Fig 7. It can be observed that a similar memory effect to sandstone occurs during cascade loading, where the displacement remains essentially unchanged from the unloading of the previous cascade to the peak stage of the current cascade. After reaching the peak load in the previous cascade, the displacement continues to increase during the loading of the next cascade. Of the three mixtures, the test block loaded with mixture 2 had more stepped loads, and the phenomenon was closer to that of sandstone.

**Fig 8:**
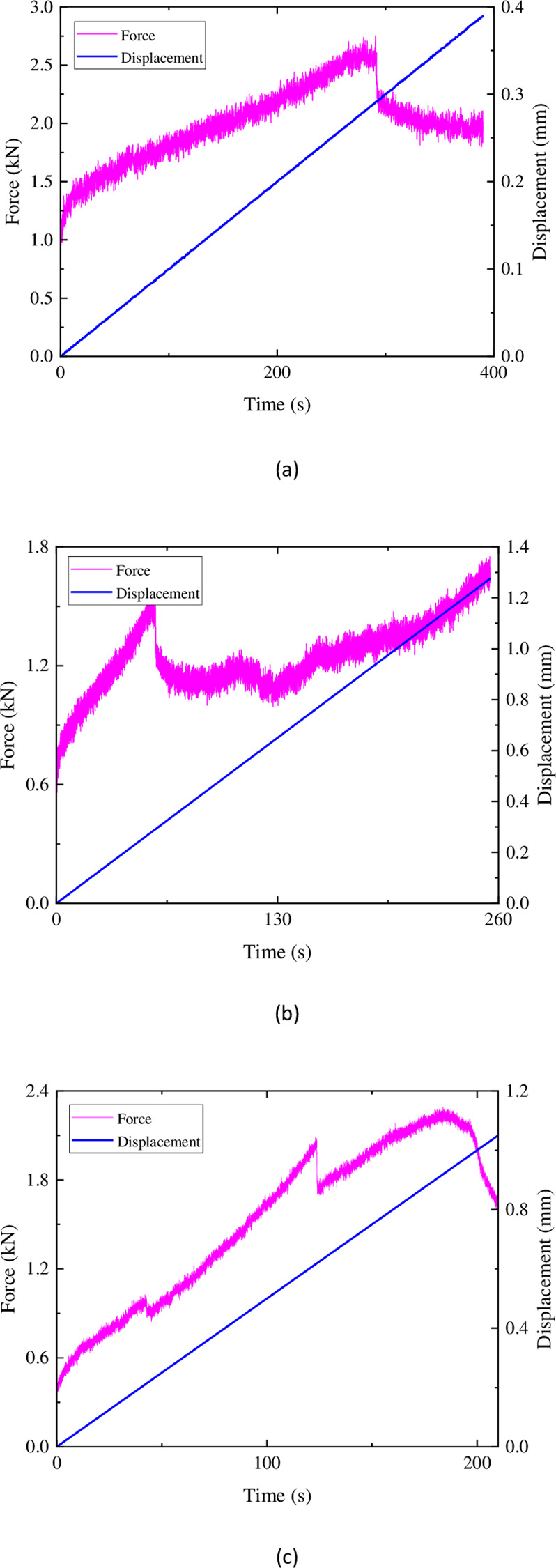
Force and displacement in sandstone uniform loading: (a) ratio 1 (b) ratio 2 (c) ratio 3.

The Brazilian Splitting Tensile Test of similar materials is shown in Fig 8 below. As the load is applied, the deformation increases linearly at a relatively constant rate. However, the load fluctuates during the loading process due to the relatively soft texture and higher porosity of the similar materials compared to sandstone. The specimen undergoes initial damage at the steel wire loading points, leading to load-bearing failure on the loading plate. Under servo control, the load fluctuates. In addition, during loading, as the load increases, the loading force undergoes sudden downward changes. This is because the specimen experiences significant damage and develops cracks but has not yet completely failed, resulting in a sudden decrease in load-bearing capacity. After the formation of cracks in the specimens shown in Fig 6(B) and 6(C), the specimens still retain some load-bearing capacity, and the load they can bear exceeds the peak value before the sudden change, until the specimens are completely destroyed. In Fig 8(A), however, after the sudden downward change in load, it continues to trend downward. This can be considered as the cracks generated in the specimen during the abrupt change have penetrated through the interior, causing the specimen to lose its load-bearing capacity, leaving only a partial cohesion force to temporarily maintain relative integrity. Compared to Mix Proportions 1 and 3, the similar materials with Mix Proportion 2 exhibit a higher similarity in the force-displacement curves during loading, resembling sandstone more closely.

### 3.2 Amplitude-frequency characteristics of acoustic emission

The amplitude-frequency characteristics of acoustic emission refer to the variation of the amplitude of the acoustic emission signal with respect to frequency. These characteristics can reflect the frequency response of acoustic wave vibrations during crack propagation and failure within rocks. When the interior of a rock is subjected to a sufficiently large load, localized stress concentration can lead to the generation of small cracks. These cracks release high-frequency acoustic wave vibrations as they propagate and merge. The frequency and amplitude of the acoustic wave vibrations can indicate the extent of crack propagation. When approaching the failure state, the crack propagation speed increases, resulting in higher frequency and larger amplitude of the emitted acoustic waves. Therefore, the amplitude-frequency characteristics are crucial for analyzing and evaluating rock mechanics experiments. By analyzing the amplitude-frequency characteristics of acoustic emission signals, one can determine the characteristic frequencies and precursory signals of rock failure, enabling the assessment and prediction of failure mechanisms and strength.

**Fig 9:**
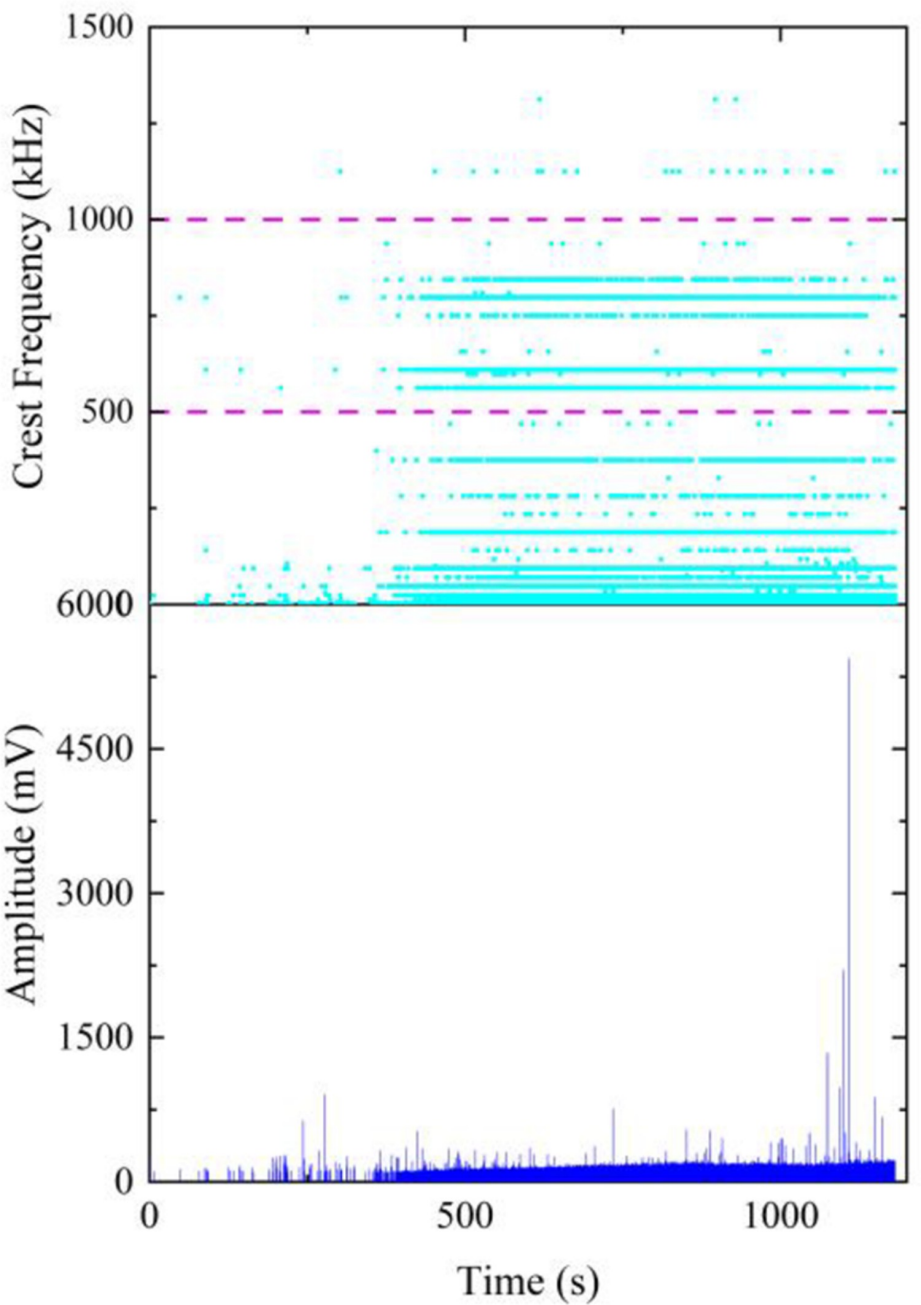
Amplitude-frequency characteristics of sandstone acoustic emission.

The amplitude-frequency data of sandstone acoustic emission is shown in Fig 9. It can be observed that the peak frequencies exhibit a banded distribution along the time dimension within the range of 0 kHz-1500 kHz. The peak frequencies are categorized into low (0 kHz-500 kHz), medium (500 kHz-1000 kHz), and high (1000 kHz-1500 kHz) intervals based on their numerical values. The peak frequencies are concentrated in the low-frequency range, followed by the medium frequencies, with fewer high-frequency components. High peak frequencies are also present during periods of high amplitude values, while low amplitude values and low peak frequencies are observed throughout the loading process. These observed amplitude-frequency characteristics align with the rock failure process, where microcracks play a primary role. Numerous low-frequency and low-amplitude acoustic emission signals are generated due to the presence of these microcracks. As the microcracks propagate and develop, they gradually connect, forming larger cracks and generating high-frequency and high-amplitude acoustic emission signals. Ultimately, the development of large cracks leads to macroscopic failure of the rock

**Fig 10:**
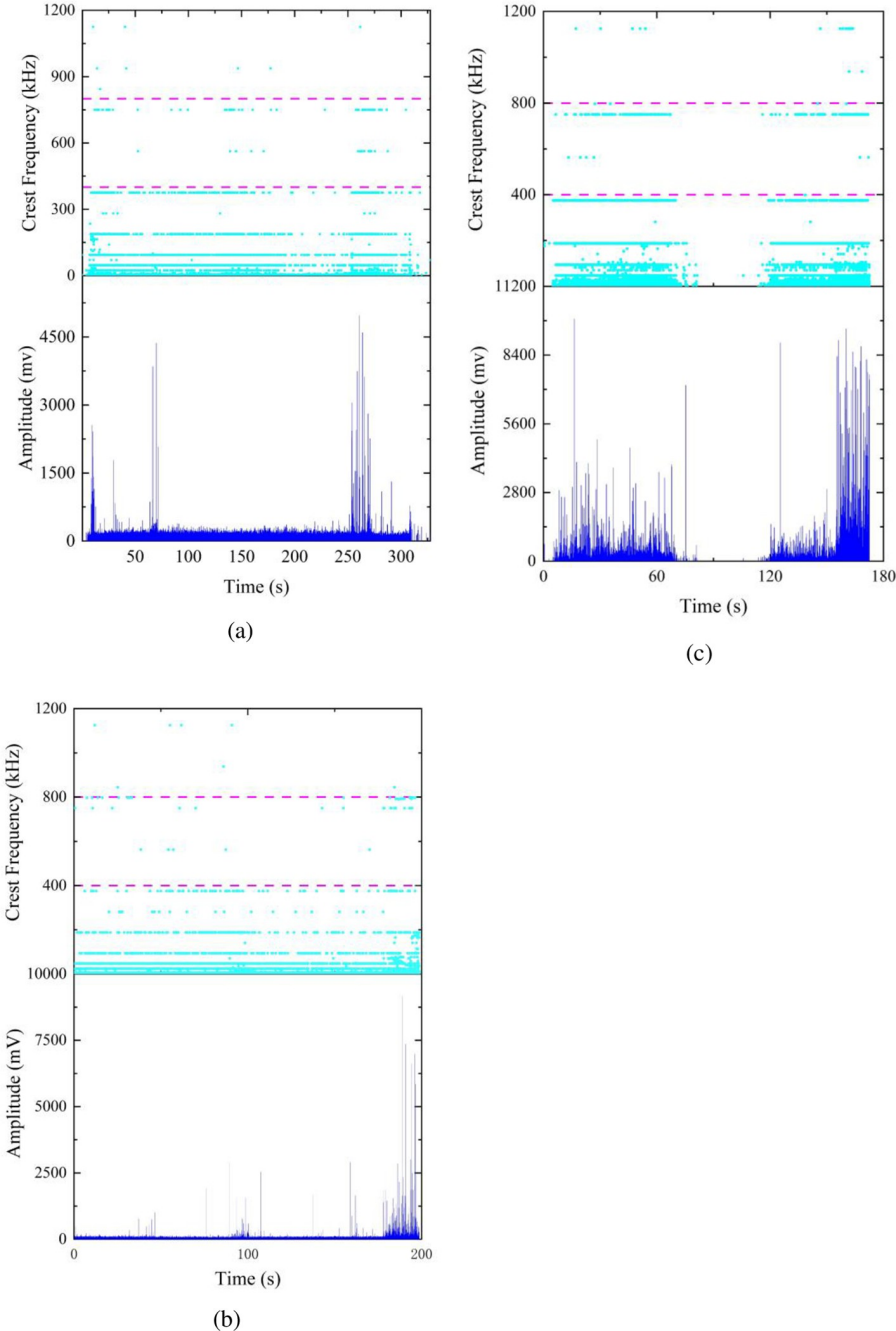
Acoustic emission amplitude-frequency characteristics of similar materials: (a) ratio 1 (b) ratio 2 (c) ratio 3.

The amplitude-frequency characteristics of the similar material exhibit similarities to sandstone. The peak frequencies also display a banded distribution across the low, medium, and high frequency ranges, with higher amplitude values mainly concentrated in the initial and near-failure stages. In Fig 10(A), an initial loading stage generates an amplitude of 2600mv, followed by intermittent occurrences of higher amplitudes within the subsequent 50 seconds. The highest peak amplitude during this stage appears at 61 seconds, after which the amplitude values stabilize at a lower level. Between 250s and 265s, high amplitude values are concentrated, surpassing the maximum amplitude of the initial stage. The amplitudes then trend back to a stable, lower level until the final failure of the specimen. In Fig 10(B), high amplitude values sporadically occur during the loading process and become more concentrated in the near-failure stage. In Fig 10(C), amplitudes are mainly concentrated within two time intervals: 0s-75s and 120s-180s, with high amplitude values concentrated in the initial loading and near-failure stages. However, unlike sandstone, the similar material demonstrates a reduced distribution of peak frequencies in the medium-frequency range, and there is a higher presence of high-amplitude values compared to sandstone.

In summary, the similar material exhibits similar amplitude-frequency characteristics to sandstone, with peak frequencies predominantly in the low to medium frequency range and higher amplitude values primarily distributed in the early loading and near-failure stages. The only difference is that the similar material has a lower proportion of peak frequencies in the medium-frequency range compared to sandstone. From Fig 10, it can be observed that, in comparison to mix 1 and mix 3, mix 2 of the similar material demonstrates amplitude-frequency characteristics that are more similar to sandstone.

### 3.3 Acoustic emission ringing count-b value characteristics

The b-value of acoustic emission can be calculated using the G-R formula. The calculation formula is given by Eq (1):

lgN=a−bm (1)

where m represents the magnitude of the earthquake, N is the number of earthquakes within the range of Δm, and a and b are constants.

This method categorizes rock fracture behavior as a type of microseismic activity, introducing the earthquake magnitude and frequency-related parameter, b-value, to analyze the rock’s damage and failure process. The magnitude of the b-value is related to the stress state and degree of fracture in the rock. When the degree of fracture is large and the internal damage in the rock is severe, the proportion of high-amplitude acoustic emission signals is relatively higher, resulting in a lower b-value. Therefore, the variation trend of the acoustic emission b-value during the loading process can be analyzed to study the rock’s damage and fracture initiation process.

In practical calculations, the entire experimental process is divided into n equal consecutive time intervals based on a time series. A reasonable time step T is determined, and each time interval of length T is treated as a data set. The seismic magnitude information, M, is calculated and recorded for each time interval using Eq (2):

$$M=A_{db}/20$$ (2)

where M represents the magnitude and A_db_ is the amplitude of the acoustic emission.

Each time interval is divided into gradient intervals [Mmin, Mmax], with the gradient value ΔM. The acoustic emission frequency, N, is calculated for each time interval. By applying the G-R formula and taking the logarithm of both the magnitude and frequency, a linear fit can be performed to obtain the acoustic emission b-value for that time interval

**Fig 11:**
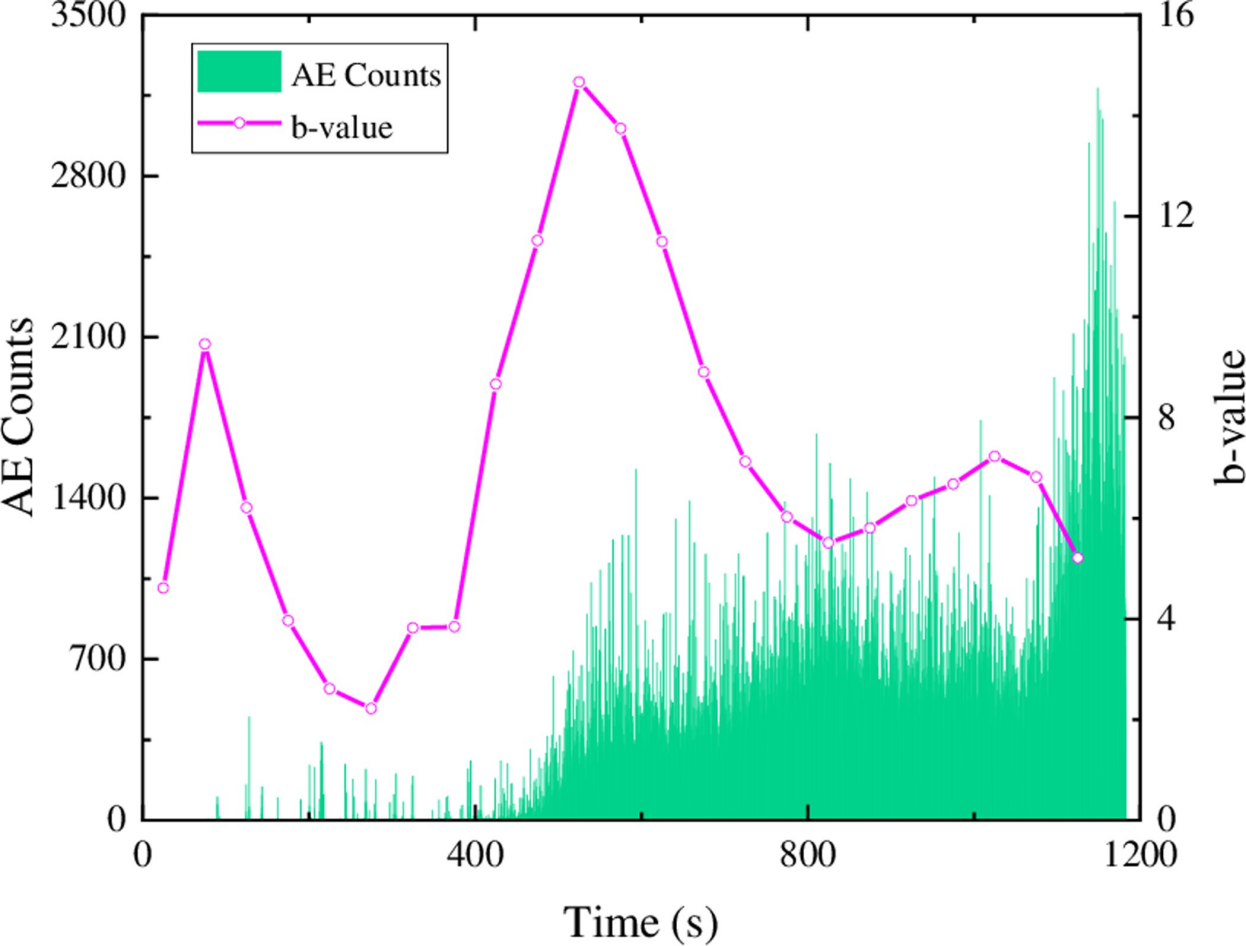
Characteristics of acoustic emission ringing count-b value of sandstone.

In Fig 11, during the initial loading stage of the sandstone specimen, the stress on the rock is relatively low, resulting in fewer ringing counts and a higher b-value of acoustic emission. This indicates that there is minimal development of microcracks during the initial loading phase. As the stress continues to increase, the internal microcracks in the rock gradually develop and expand. This leads to an increase in the number of acoustic emission events, resulting in an increasing trend of ringing counts and a rapid decrease in the b-value. This suggests that as the stress increases, the microcracks inside the rock develop into larger cracks. Subsequently, the ringing counts decrease while the b-value slowly increases, indicating that microcrack development continues to be the primary mechanism. When the ringing counts increase again, the b-value shows a decreasing trend, indicating that the internal microcracks further develop into larger cracks. In the subsequent process, the microcracks continue to expand, leading to the overall connectivity of cracks and the complete failure of the rock. The rapid decrease in the b-value after it reaches a peak also indicates that the cracking has led to macroscopic failure of the rock.

**Fig 12:**
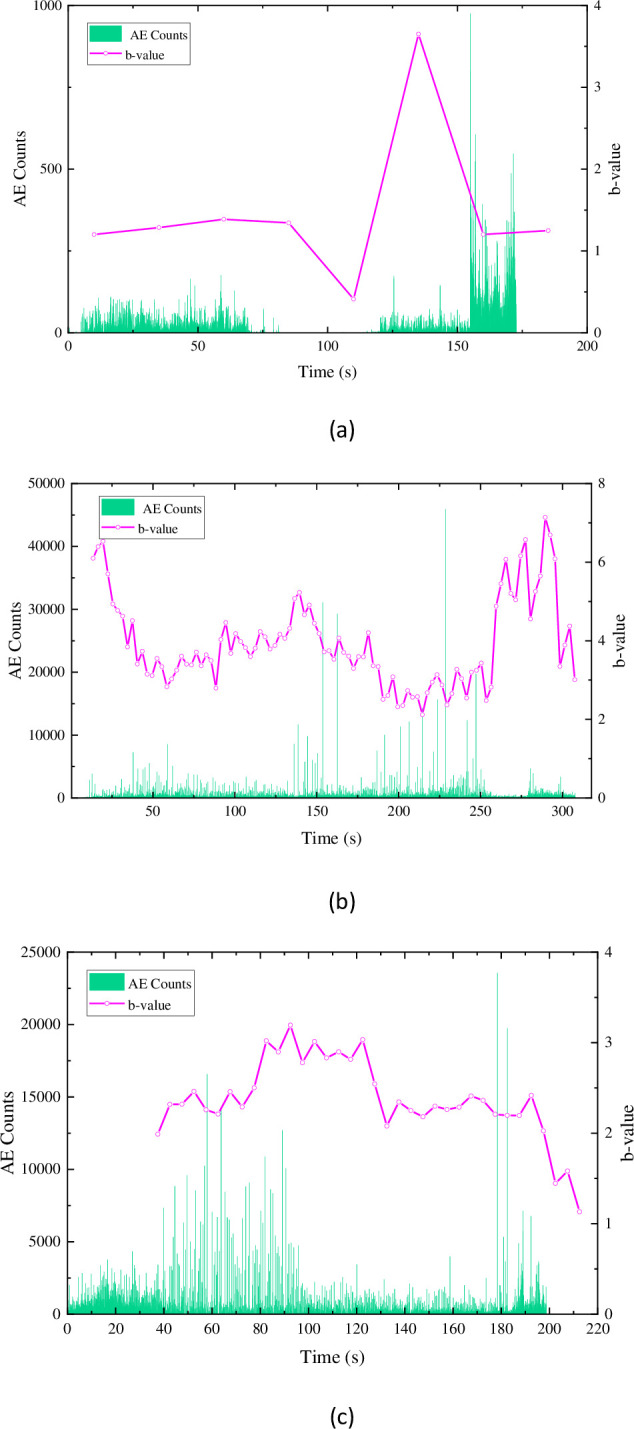
Acoustic emission ringing count-b value characteristics of similar materials: (a) ratio 1 (b) ratio 2 (c) ratio 3.

In Fig 12, it can be observed that the AE counts of similar materials are relatively sparse compared to sandstone in terms of their temporal distribution. However, except for the composition 1 (Fig 12(A)) specimen, the magnitude of AE counts is higher than that of the sandstone material. This is presumed to be due to the generation of more microcracks during the failure process of the similar materials, leading to a higher number of AE counts. This presumption is also reflected in the values of the b-value. The maximum b-value for the sandstone material is 14.67, with an average of 7.06. However, the maximum b-value for the three compositions of the similar materials are only 7.13 (composition 2), with average values of 1.46, 3.94, and 2.38, respectively. It can be seen that during the failure of similar materials, the development of fractures is mainly dominated by microcracks. In the early to middle stage, microcracks gradually expand into larger cracks, and in the middle to later stage, more microcracks are generated and eventually lead to complete failure of the specimen.

Based on the distribution of AE counts and b-values, it can be concluded that composition 2 of the similar materials exhibits a better similarity in the development and expansion of fractures compared to sandstone.

### 3.4 Rupture point evolution based on acoustic emission energy

The occurrence of acoustic emission phenomenon is accompanied by the release of energy. In the process of acoustic emission monitoring, spatial positioning techniques can be used to capture the rupture points of rocks. By overlaying the coordinates of the captured rupture points with the released energy values, a three-dimensional representation can be reconstructed to depict the energy distribution during the rock fracture process. As shown in the figure below, the color of the spheres represents the energy released at the rupture points. The color gradient from green to red indicates increasing energy values, and the size of the spheres corresponds to the energy values.

**Fig 13:**
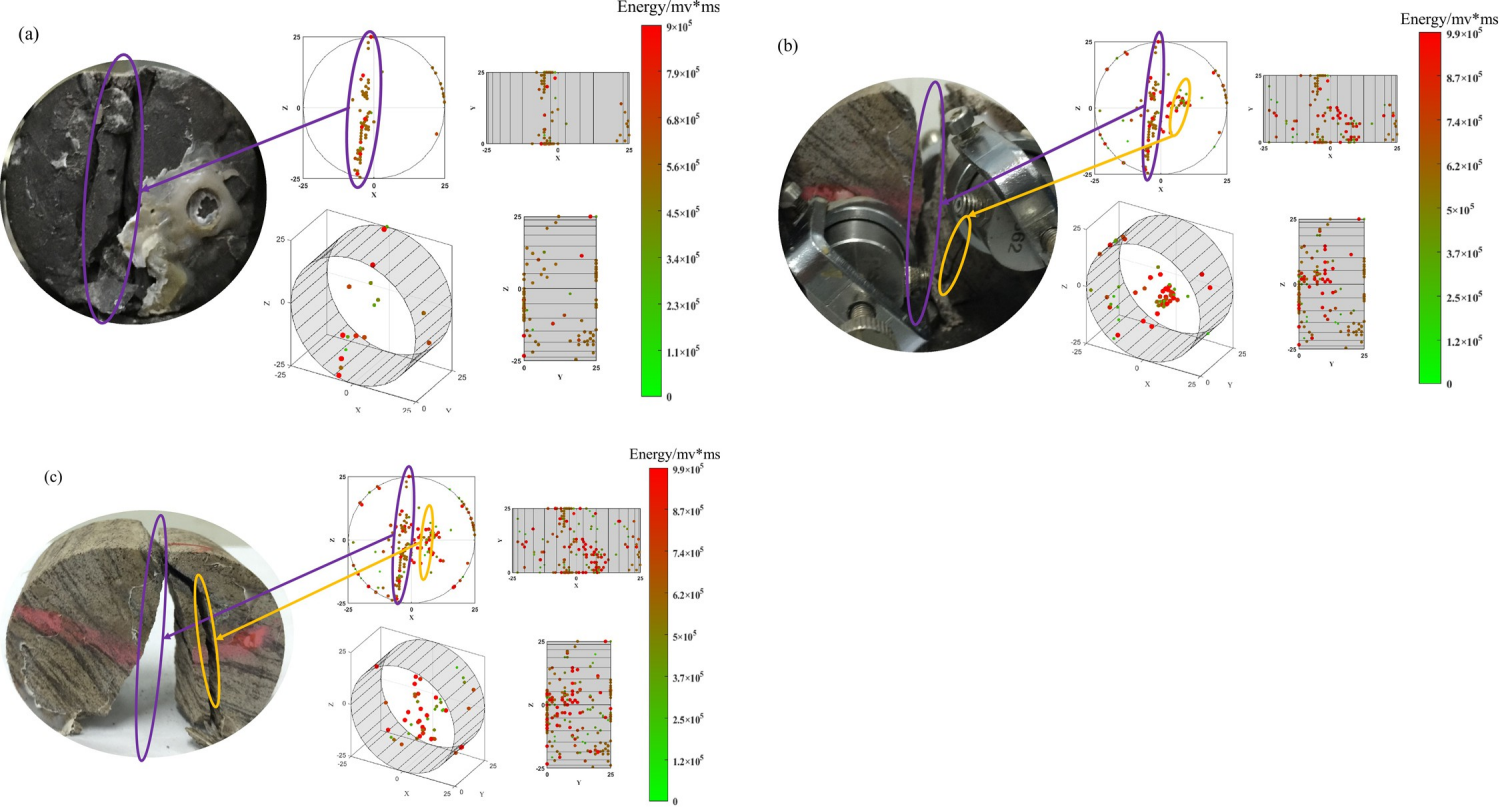
Inversion of sandstone fracture point based on acoustic emission energy.

In Fig 13, it can be observed that the cracks in the sandstone mostly traverse the specimen radially, slightly deviating from the axial center. The acoustic emission monitoring reveals that the rupture points are concentrated at the splitting cracks, and secondary fissures are developed in all three specimens at the splitting sites. In Fig 13(A), the secondary cracks result in the formation of elongated fragments at the splitting site, while Fig 13(B) and 13(C) show secondary fissures penetrating the specimens. This can also be observed in the acoustic emission inversion map, where rupture points are clustered at the locations of secondary fissures (indicated by yellow circles in the figure). Acoustic emission monitoring indicates that rupture points are mostly concentrated at the ends of the specimens and near the end faces, suggesting active crack development at the end faces, resulting in a higher occurrence of acoustic emission events. It can be inferred that in the process of splitting failure, cracks develop and propagate more extensively near the end faces, eventually leading to the failure of the specimens.

**Fig 14:**
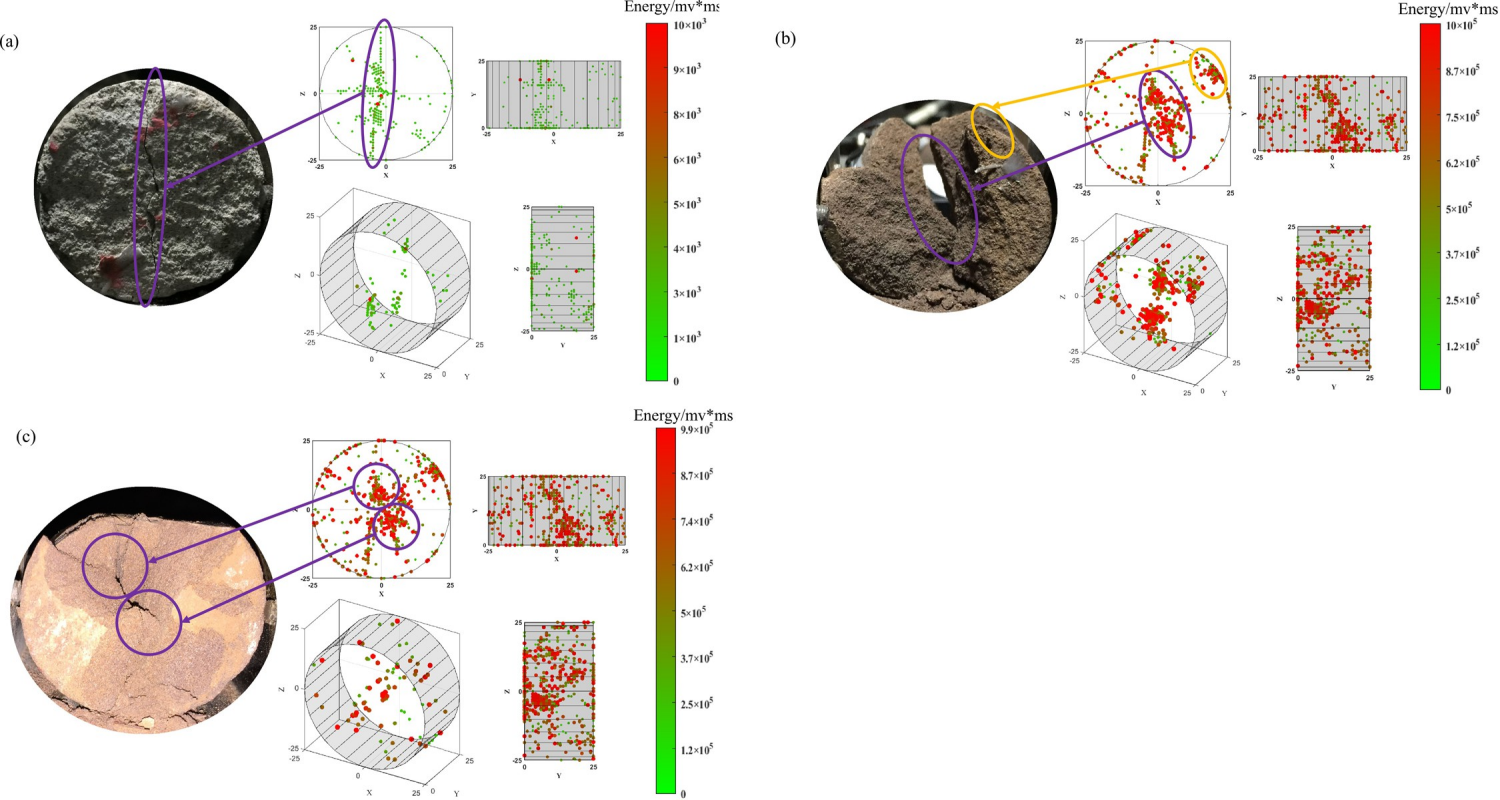
Fracture point inversion of similar materials based on acoustic emission energy: (a) ratio 1 (b) ratio 2 (c) ratio 3.

In Fig 14, it can be seen that the number of rupture points detected by acoustic emission monitoring in the specimens of similar materials is significantly higher than in the sandstone specimens. In Fig 14(A), the energy of the rupture points is relatively low, and the three-dimensional representation shows that the rupture points mostly cluster near the splitting cracks close to the end faces. The specimen exhibits a nearly “S”-shaped splitting crack along the radial direction without obvious secondary fissures. In Fig 14(B), in addition to the splitting crack, a large number of rupture points are detected near the axial center. Comparing it with the reference specimen, it can be observed that the specimen experiences local fragmentation at the rupture point cluster, which is consistent with the failure phenomenon of the sandstone. In Fig 14(C), the splitting crack does not completely penetrate the specimen. Fragmentation occurs at the upper loading location, and the crack penetrates the specimen in the lower left part, while localized fragmentation appears near the upper left and lower right regions close to the axial center.

### 3.5 Rupture point evolution based on time-space

Based on time series, three-dimensional inversion mapping of rupture point coordinates can be used to obtain the spatial distribution of rupture points along the temporal process, i.e., the spatiotemporal evolution of rupture points. The spatiotemporal evolution reveals the crack propagation process. As shown in the figure below, the distribution of rupture points at 20%, 40%, 60%, 80%, and 100% of the total loading time is plotted with an interval of 20% from the start time of 0 seconds.

**Fig 15:**
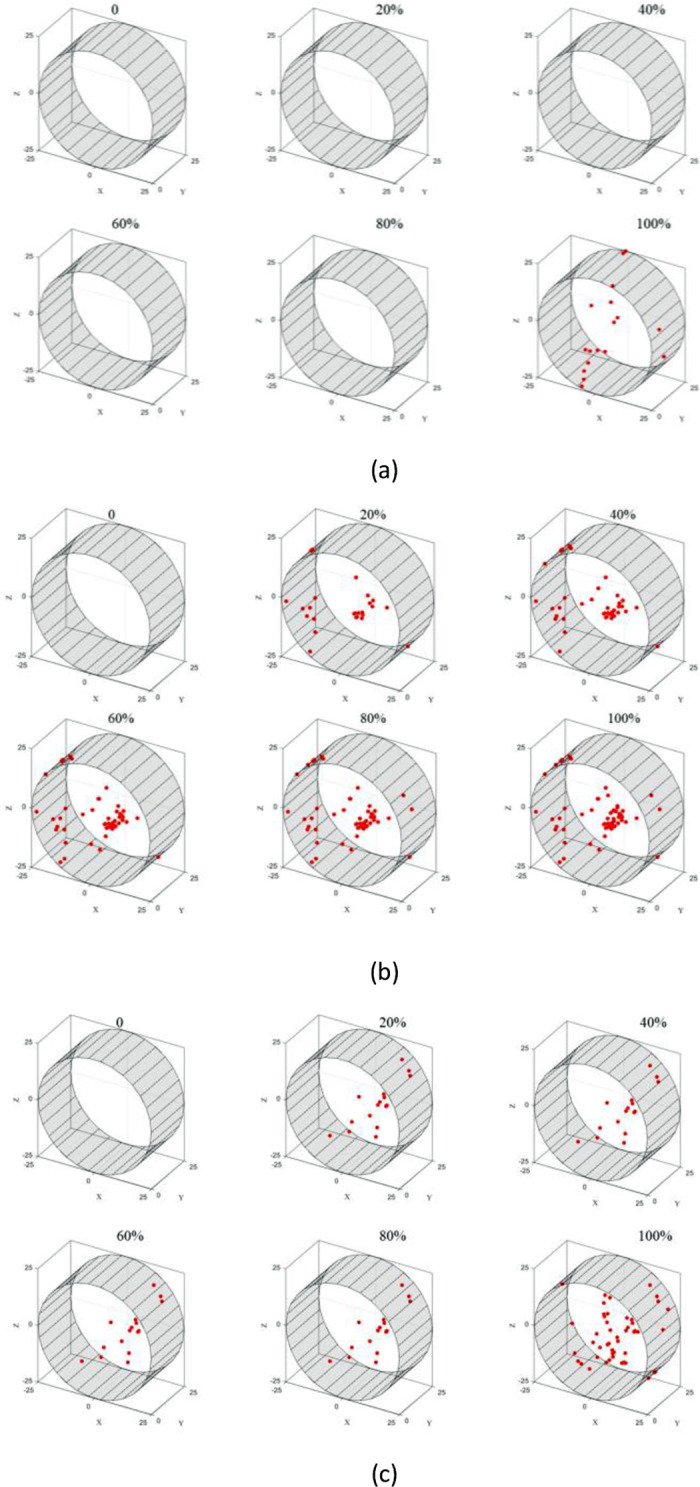
Spatio-temporal evolution of fracture point of sandstone test block.

In Fig 15(A), it can be observed that no acoustic emission rupture points are detected until the loading reaches 80%. It wasn’t until the end of the loading process that rupture points were detected, distributed along an approximate straight line near the end face in the Z-axis direction. This indicates that during the loading process, there were few cracks generated in the test block until just before its peak tensile strength was reached, at which point rapid crack expansion and penetration caused the block’s failure.

In Fig 15(B), it can be seen that the specimen starts to crack at 20% of the loading and the crack initiation point is located slightly above and to the right of the center. With further loading, rupture points continue to accumulate at that location, gradually forming a vertically connected main crack towards the left of the center.

In Fig 15(C), it can be observed that a significant number of rupture points are already concentrated in the lower right region slightly below the center at 20% loading. As the loading progresses, more rupture points are generated at this location. At 100% loading, some rupture points appear in the upper left region, indicating that the specimen initiates cracking from this area and the cracks continue to propagate during loading, eventually forming a rupture crack that penetrates the center.

Overall, it can be seen that the sandstone specimen mostly initiates cracking from the center and the resulting rupture cracks either pass through the center or slightly deviate from it. Secondary cracks near the center area are likely to cause localized damage in the center region.

**Fig 16:**
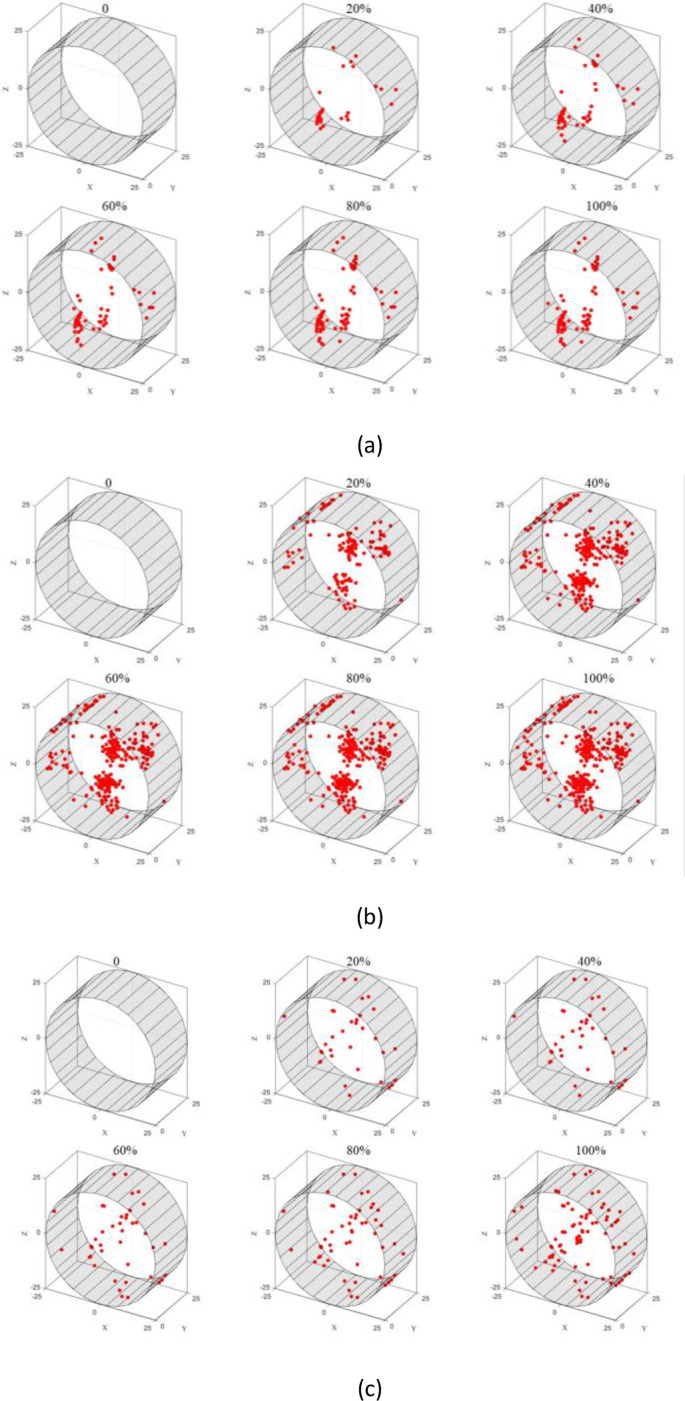
Spatial and temporal evolution of fracture point of similar material test block: (a) ratio 1 (b) ratio 2 (c) ratio 3.

In Fig 16(A), it can be seen that the similar material specimen with mix ratio 1 exhibits rupture points distributed in a linear pattern along the Z-axis in both end faces at 20% loading. As the loading progresses, more rupture points appear near these two lines, indicating that the specimen undergoes cracking along a straight line that passes through the center of the end faces.

In Fig 16(B), a significant number of rupture points are generated as soon as the loading reaches 20%. They are mainly distributed in the upper left, lower right, and upper right regions of the specimen. With further loading, more rupture points accumulate in these three areas, indicating that the specimen initiates cracking at the center and the failure mainly occurs at the center and the upper right region. This is consistent with the failure photo of the specimen shown in Fig 14.

In Fig 16(C), rupture points start to appear from the center at 20% loading and gradually increase, forming an inclined "Y"-shaped distribution, ultimately resulting in destructive fractures along the diagonal direction. The photo of the specimen in Fig 14 also shows that the specimen did not undergo longitudinal through-thickness splitting.

In conclusion, in the Brazilian tensile test of similar materials, the specimen cracks from the center near both end faces. However, due to different material ratios, there are differences in the subsequent cracking process. Considering the secondary damage near the center during the failure process of the sandstone specimen, the spatiotemporal evolution of mix ratio 2 is more similar to that of sandstone.

## 4 Discussions

In the research on Brazilian Splitting Test, scholars mostly use rock materials as objects of study and fewer experiments are conducted using similar materials. Therefore, there are relatively few research results on the selection and proportioning of similar materials in this aspect. In the loading modes of Brazilian splitting in Brazil, displacement or force control is generally chosen. In this study, the commonly used incremental loading mode in compression tests was introduced to conduct Brazilian splitting tests on sandstone and similar materials.

During the experiments, several similarities were observed between the sandstone specimens and the similar material specimens under both loading modes. In the incremental loading, both the sandstone and similar materials exhibited a memory effect. In the constant rate loading, the relationship between force and displacement in the later stage of loading was approximately linear for the sandstone specimens, while it was closer to a linear relationship in the similar material with the proportioning of 2.

In terms of acoustic emission characteristics, in the amplitude-frequency distribution, the amplitude of the sandstone was relatively uniform, with high amplitudes appearing near the failure stage. The similar material with the proportioning of 2 showed a similar amplitude distribution compared to the sandstone. In the acoustic emission resonance technique (b-value), the characteristics of the sandstone were consistent with the research reports of scholars, while the similar material with the proportioning of 2 was closer to the characteristics of sandstone.

In terms of failure characteristics and spatio-temporal evolution, the sandstone specimens cracked from the center of both end faces, and the cracks penetrated the center of the specimens radially, partially deviating from the center. Near the center, local damage occurred in association with the generation of secondary cracks. This is similar to the findings of previous research by scholars. The similar materials showed similar cracking initiation at the center of both end faces, but different phenomena were observed during subsequent crack propagation. Only the specimens with the proportioning of 2 exhibited through-cracks and localized damage caused by the development of secondary cracks at the center.

## 5 Conclusion

This article is based on Brazilian splitting experiments and employs two loading methods, namely incremental loading and constant-rate loading, to study the destructive characteristics and selection of sandstone and various proportionally similar materials under tensile stress. The following conclusions were drawn:

1. In incremental loading, both sandstone and rock-like materials exhibit a memory effect and undergo plastic deformation. In constant-rate loading, after the compaction stage, the relationship between force and displacement shows an approximate linear trend for both materials.
2. The peak frequency of acoustic emission in both sandstone and rock-like materials follows a banded distribution. Sandstone’s peak frequency concentrates in the mid to low-frequency range, while rock-like materials mainly focus on the low-frequency range. As cracks generate, develop, and lead to failure in specimens, the acoustic emission signals gradually transition from low-frequency and low-amplitude to high-frequency and high-amplitude. High-frequency and high-amplitude signals primarily concentrate in the proximity of the failure stage, serving as precursors to material failure.
3. The b-values of acoustic emission in sandstone exhibit an approximate "W" shape distribution, decreasing again during final failure, with AE counts being inversely related to b-values. Rock-like materials have slightly higher AE counts than sandstone, and their b-values are relatively lower.
4. Both sandstone and rock-like materials initiate cracking from the center of the two end faces, with local damage induced by secondary cracks near the main crack. The failure characteristics of rock-like materials are essentially consistent with those of sandstone.

In summary, the rock-like materials with three selected ratios are similar to sandstone in terms of mechanical loading, acoustic emission characteristics, and failure morphology. Among them, the ratio of quartz sand: cement: water = 9:1:0.9 closely resembles sandstone and can be used for similar simulated experimental studies.

The article analyzes and studies the destructive characteristics and acoustic emission features of sandstone and rock-like materials under different loading paths, providing theoretical support for understanding the rock’s failure mechanism under tensile stress and the selection of proportionally similar materials. However, the exploration of rock-like material ratios is limited to the discussed ratios, and further research is needed to determine appropriate proportionally similar compositions for rock materials other than sandstone.
